# Male-biased dominance in greater bamboo lemurs (*Prolemur simus*)

**DOI:** 10.5194/pb-11-13-2024

**Published:** 2024-03-28

**Authors:** Lilith Sidler, Johanna Rode-White, Peter M. Kappeler

**Affiliations:** 1 Department of Sociobiology/Anthropology, University of Göttingen, 37077 Göttingen, Germany; 2 Cologne Zoo, 50735 Cologne, Germany; 3 Behavioral Ecology and Sociobiology Unit, German Primate Center, Leibniz Institute of Primatology, 37077 Göttingen, Germany

## Abstract

Intersexual dominance relationships in virtually all lemur species have been reported to be female-biased. Although a claim of male dominance in greater bamboo lemurs (*Prolemur simus*) which was not supported by data is unusual against this background, it is in line with recent studies on other lemur species that suggest the existence of a continuum of intersexual dominance relationships. We therefore studied the details of agonistic interactions among adults of one captive group of *P. simus* at Cologne Zoo. This very preliminary study confirmed male-biased dominance because the adult male of the study group won all agonistic interactions with all three adult females, and the male was never dominated by any of the females. This result raises several interesting questions about the mechanisms and evolution of intersexual dominance relationships in group-living lemurs and should encourage similar future studies of additional groups of this species – ideally in the wild.

## 1 Introduction

The main evolutionary benefits of group formation are counterbalanced by the unavoidable costs of coordination and competition (de Waal, 1986; Majolo et al., 2008; Markham and Gesquiere, 2017). Because members of the same species compete for the same resources, their interactions always entail subtle or overt forms of competition (Rowell, 1974; Drews, 1993). If competition involves the exchange of aggressive and or submissive behavioral elements, these interactions are referred to as agonistic interactions (Walters, 1980). A dyadic agonistic interaction is decided whenever only one individual displays submissive behavior, irrespective of the display of aggression by the other dyad member (Pereira and Kappeler, 1997; de Vries, 1998). The direction and consistency of repeated decided agonistic interactions can be used to infer dyadic dominance relationships, which, in turn, can be arranged in a dominance hierarchy (Schjelderup-Ebbe, 1922; Sánchez-Tójar et al., 2018). In groups of primates, individuals often cluster within a hierarchy as a function of their sex, with either all or most males outranking all females or – more rarely – vice versa (Hausfater, 1975; Bernstein, 1976; Kappeler et al., 2022a).

Because male-biased sexual dimorphism is expected and widespread among mammals, the ability of male mammals to dominate females has long been regarded as an unavoidable side effect of physical superiority and greater aggressiveness (Richard, 1987; Kappeler, 1993). The much rarer opposite pattern of strict female dominance, in contrast, appeared to require special explanation and generated numerous hypotheses that typically invoked taxon-specific special factors to explain the evolution of this sex role “reversal” (Lewis, 2018, 2020; Kappeler et al., 2022b). However, recent comparative studies across primates and other mammals found that intersexual dominance relationships range across a continuum, with complete male and female dominance only representing extreme end points (Kappeler et al., 2022a, b). The long-standing notion of widespread exclusive female dominance in lemurs was previously particularly challenged by a report of complete male dominance in the greater bamboo lemur (*Prolemur simus*). Unsystematic observations of a captive group suggested that “males are dominant in this species” (Roullet, 2011), but no data were presented to support this claim. Whereas no clear sex bias in intersexual dominance relations has been reported for brown, black and rufous lemurs (Pereira et al., 1990; Digby and Kahlenberg, 2002; Roeder et al., 2002), challenging the ubiquity of female-biased dominance among group-living lemurs, a male bias in intersexual dominance has not been described for any lemur species. We therefore conducted a preliminary observational study of a group of captive *P. simus* at Cologne Zoo, Germany, to contribute the first quantitative data on intersexual dominance relationships in this elusive species.

## 2 Materials and methods

Greater bamboo lemurs live in groups of 2–27 (mean 9.7; N= 36 groups; including 1.4 adult males and 3.2 adult females; Peter M. Kappeler, unpublished data) individuals. They are cathemeral and inhabit the rain forests of Eastern Madagascar, where they feed exclusively on bamboo shoots (Ravaloharimanitra et al., 2011; Olson et al., 2013; Nazim et al., 2022). Adults weigh 2200–2500 g and are exceptional among lemurs in that males are on average 13 %) heavier than females (Kappeler et al., 2019). There is no long-term study population and only a few behavioral studies of this Critically Endangered species have been conducted (Mihaminekena et al., 2019; Ravaloharimanitra et al., 2020).

The study group at Cologne Zoo consisted of six individuals: one adult male (19 years), three adult females (11, 9 and 6 years) and two juvenile males 2 and 3 years old). The oldest female was the mother of the two females and the juveniles; the adult male was the father of all individuals, including the oldest female. One of us (Lilith Sidler) collected behavioral data during the non-reproductive season in June and July 2022. All individuals served as focal animals, and one sampling session lasted up to 1 h. In total, we collected 112 h of data across 21 d (75 h on adult focal animals). The animals had two indoor compartments and two outdoor compartments available. Each indoor compartment was 5 m × 3.50 m = 17.50 m^2^ (total = 35 m^2^) and each outdoor compartment was 5 m × 2.50 m = 12.50 m^2^ (total 25 m^2^). The enclosures are approx. 4 m high. Both indoor and outdoor areas are separated from visitors by a complete glass panel, so that the focal animals could always be seen by the observer. The four sub-compartments provided the animals with opportunities to move away and to hide.

We classified agonistic behaviors as either aggressive (bite, charge, chase, hit, hit–push, feint-to-hit, grab, growl, lunge, stalk) or submissive (cower, being displaced, flee, jump away, glances, chirp), following definition in the ethogram of ring-tailed lemurs (Pereira and Kappeler, 1997), the sister genus of bamboo lemurs (Everson et al., 2023). High-pitched chirping often accompanied other submissive behaviors and was therefore regarded as a species-typical submissive signal. After each agonistic interaction, defined as the exchange of at least one aggressive or submissive behavioral element, we assigned an A (aggressive), S (submissive), AS or O (other/non-agonistic) to both members of the dyad based on the behaviors they displayed. We regarded dyadic interactions where only one individual exhibited submissive behavior as decided conflicts and all other conflicts as undecided. We calculated the rate of conflicts, the proportion of decided conflicts, the proportion of conflicts with submissive signals and the ratio of aggressive behaviors to submissive behaviors for each possible sex combination of dyads, omitting interactions among juveniles. We generated a winner–loser matrix based on all decided male–female and female–female conflicts and used it for the calculation of David's score and I&SI for the computation of a dominance hierarchy, using the functions “DS” and “ISI” in the R package “EloRating” (Neumann et al., 2011) as well as an index of hierarchical transitivity with the function “transitivity” of the same package.

No ethical consent was required for this study, as no animals had to be trapped or handled.

## 3 Results

The adult male of the study group won all agonistic interactions with all three females, and the male was never dominated by any of the females (Table 1). The vast majority of inter- and intrasexual conflicts were decided and involved submissive behaviors (Table 2). The male displayed aggressive behavior in only 15 % of conflicts, and 87 % of female submissive behavior was displayed in the absence of aggression from the male. The male never displayed any submissive behaviors. There was no noticeable difference between the mean inter- and intra-sexual conflict rates, but the variation was much higher in male–female conflicts. The highest rate of female–female conflicts was 6 conflicts per hour and that of male–female interactions 14 conflicts per hour. No polyadic agonistic interactions were observed. Both ISI ranks and David's score generated a hierarchy with the male at the top, followed by the females ranked from oldest to youngest (normDS: M – 3.0; F1 – 2.0; F2 – 0.9; F3 – 0.1). There were no unknown relationships. The dominance hierarchy had a transitivity of Pt = 1, indicating complete transitivity.

**Table 1:** Winner–loser matrix of agonistic interactions of a captive group of *Prolemur simus* in Cologne Zoo, Germany. AM: adult male; AF: adult female (numbers: age, from oldest to youngest).

		Loser			
		AM	AF1	AF2	AF3
Winner	AM	–	42	77	29
	AF1	0	–	36	24
	AF2	0	0	–	4
	AF3	0	0	0	–

**Table 2:** Summary of dyadic agonistic interactions of a captive group of *Prolemur simus* in Cologne Zoo, Germany. OT: observation time in hours; Rate: mean conflict rate per hour and dyad; % decided: percentage of decided conflicts; % sub: percentage of conflicts with submissive behaviors; Ratio A : S: ratio of aggressive to submissive behaviors.

	N conflicts	N dyads	OT	Rate	% decided	% sub	Ratio A : S
Total	216	6	75	0.5±0.6	98.1	99.1	0.23
FF	65	3	56	0.5±0.6	98.5	98.5	0.27
MF	151	3	75	0.6±1	98.0	98.7	0.16

% decided conflicts won by male: 100.

## 4 Discussion

The results of this preliminary study confirm the assertion that *P. simus* males can indeed dominate all females in their group. Unlike females in all other lemur species studied to date (Kappeler et al., 2022b), *P. simus* females were unable to elicit any submissive behavior in the adult male. More precisely, whereas females of some *Eulemur* and *Microcebus* species did not win the majority of their agonistic interactions with males (Fig. 2 in Kappeler et al., 2022b​​​​​​​), they were nonetheless able to elicit submissive behavior from males in some conflicts or dyads. While the present study was of relatively short duration and involved only a single group with a single male, similar studies of other lemur species with comparable limitations (e.g., *Daubentonia*: Rendall, 1993; *Eulemur*: Marolf et al., 2007; Digby and Kahlenberg, 2002; *Microcebus*: Hohenbrink et al., 2015; *Propithecus*: Ramanamisata et al., 2014; *Varecia*: Raps and White, 1995; Meyer et al., 1999) yielded robust insights into intersexual dominance relations, as revealed by subsequent studies of the same taxa (reviewed in Kappeler et al., 2022b). A further unavoidable shortcoming of the present study is due to the fact that the adult male was the father of two of the females, emphasizing another preliminary aspect of our study. Because indices of intersexual dominance tend to be variable across groups and within groups over time (Balasubramaniam et al., 2012; Kappeler et al., 2022a; Neumann and Fischer, 2023), future studies of additional groups of *P. simus* may reveal more variation in intersexual dominance than the present one.

Thus, our study supports the insight that sex-biased dominance hierarchies among lemurs are variable among species and not invariably biased towards females, challenging the universal validity of most existing hypotheses about the evolution of female dominance among lemurs (summarized in Kappeler and Fichtel, 2015; Lewis, 2020) and supporting more recent notions about determinants of sex-specific dominance (Davidian et al., 2022). From a proximate perspective, it is worth pointing out that *P. simus* is the only lemur species in which males are on average more than 10 % heavier than females (Taylor and Schwitzer, 2011), affording males a physical advantage in agonistic interactions with females. Such a sex bias in sexual size dimorphism has only been reported for one other species of Malagasy mammal (*Tenrec ecaudatus*; Kappeler et al., 2019), underscoring the question of why sexual dimorphism and intersexual dominance of all lemur species are only male-biased in *P. simus*.

This very preliminary study of only one captive group does not allow any firm conclusions. Yet, it indicates some potentially interesting aspects of the social structure of this species that should now be replicated in other study populations. If confirmed by future studies, the present results have implications for understanding this – for lemurs – exceptional pattern of intersexual dominance.

First, as in ring-tailed lemurs (*Lemur catta*; Pereira and Kappeler, 1997), both male–female but also female–female dyads appear to rely primarily on spontaneous submission to regulate their conflicts. If confirmed in studies of additional groups, these observations may indicate that the use of more submissive signals and fewer aggressive acts may not only characterize societies where female dominance prevails (Kappeler et al., 2022a) but that such a pattern is perhaps more characteristic of lemurs, irrespective of their pattern of intersexual dominance.

Second, greater bamboo lemurs are the only lemur species with a noticeable male-biased sexual size dimorphism (Kappeler et al., 2019). Because male dominance has traditionally been linked to male physical superiority, this species may support the notion that the proximate determinants of dominance relations can trump other factors, like those to related to other aspects of intrasexual competition that have been invoked to explain sex biases in intersexual dominance. It would now be very interesting to investigate the mating system of this species to explore the potential basis of their sexual dimorphism.

Finally, if confirmed, male dominance in greater bamboo lemurs would also raise interesting questions about its evolution. If female dominance is indeed ancestral for lemurs (Lewis et al., 2023), why might these bamboo lemurs or their ancestors have lost it? Why have the environmental factors and their eco-physiological consequences that have been implicated in general discussions of the evolution of female dominance (reviewed in Kappeler and Fichtel, 2015) not had the same effects in this species? If, alternatively, components of the mating system are important predictors of male dominance (Davidian et al., 2022; Huchard et al., 2024), why might that of greater bamboo lemurs differ so fundamentally from that of so many other lemur species? We hope that field studies of known individuals of *P. simus* will be initiated before this critically endangered species has gone completely extinct after already suffering massive reductions in their geographical range in historical times (Wright et al., 2008).

## Data Availability

The raw data are made available upon reasonable request to the corresponding author.
